# miR-145 downregulates the expression of cyclin-dependent kinase 6 in human cervical carcinoma cells

**DOI:** 10.3892/etm.2014.1765

**Published:** 2014-06-06

**Authors:** JING ZHANG, LU WANG, BAOLI LI, MANPENG HUO, MINGTAO MU, JUNJUN LIU, JIMING HAN

**Affiliations:** Department of Clinical Medicine, Medical College of Yan’an University, Yan’an, Shaanxi 716000, P.R. China

**Keywords:** cervical carcinoma, miRNA-145, eukaryotic expression vector, cyclin-dependent kinase 6

## Abstract

The present study aimed to investigate the effect of the inhibition of miR-145 on cyclin-dependent protein kinase 6 (CDK6) and the proliferation of human cervical carcinoma cells. The miR-145 sequence was synthesized and cloned into pcDNA™6.2-GW to construct the recombinant plasmid pcDNA6.2-GW-miR-145. HeLa cells were divided into the micro (mi)R-145, normal control and blank groups. The transcription levels of miR-145 and CDK6 were detected using quantitative polymerase chin reaction and western blot analysis was used to examine the CDK6 protein expression. In addition, the inhibitory effect of miR-145 on the proliferation of HeLa cells was measured by an MTT assay. The recombinant plasmid pcDNA6.2-GW-miR-145 was successfully constructed and used to transfect the HeLa cells in the MiR-145 group. The miR-145 expression level in the miR-145 group was significantly higher than that in the blank group. The CDK6 expression level in miR-145 group was significantly lower than that in the blank group. Furthermore, miR-145 inhibited the proliferation of HeLa cells. In conclusion, miR-145 overexpression suppresses the expression of CDK6 and inhibits the proliferative ability of HeLa cells.

## Introduction

Currently, the incidence of cervical cancer is second only to that of breast cancer, being the second most prevalent female malignancy worldwide (1). The morbidity and mortality rates of cervical cancer patients are gradually increasing, and the disease is demonstrating a marked tendency to occur in individuals at younger ages (2). Surgical treatment is normally used at the early stages, while radiotherapy is mainly applied for patients at the advanced stages. Furthermore, cervical cancer has a high recurrence rate (3). Therefore, the elucidation of the molecular pathogenesis of cervical cancer is likely to be conducive for the diagnosis and treatment of the disease.

micro (mi)RNA are a class of endogenous non-coding RNA with only 19–21 nucleotides. miRNA participates in the regulation of numerous biological functions, including cell cycle, proliferation, differentiation or apoptosis, by degrading its associated proteins or inhibiting their expression through downstream target genes at the post-transcriptional level (4). Previously, the abnormal expression of miRNA has been found in cervical cancer, including miR-214 (5), miR-21 and miR-143 (6). These miRNAs may be involved in cervical cancer proliferation, cycle or invasion, metastasis and other tumorigenic processes. miR-145 has an important role as a tumor suppressor gene, with low expression in esophageal (7), bladder (8), colorectal and numerous other human cancer types, and affects the biological functions of tumor cells by regulating the expression of numerous downstream genes. However, to the best of our knowledge, no study has reported the role of miR-145 in cervical cancer until now. In the present study, an miR-145 expression vector was constructed using the eukaryotic expression vector pcDNA™6.2-GW, and was transfected into HeLa cervical cancer cells to examine the regulatory effect of miR-145 on its downstream target gene cyclin-dependent protein kinase 6 (CDK6).

## Materials and methods

### Plasmids, cells and reagents

The eukaryotic expression vector pcDNA6.2-GW, *E. coli* DH5α and the HeLa cervical cancer cell line were provided by the Department of Cell Biology and Genetics of the Xi’an Jiaotong University Health Science Center (Xi’an, China). The reverse transcription kit (PrimeScript^®^ RT) and SYBR Premix Ex Taq^TM^ II were purchased from Takara Bio Inc. (Dalian, China). The transfection reagents were purchased from Roche (Basel, Switzerland). TRIzol was purchased from Invitrogen Life Technologies (Carlsbad, CA, USA). Radio-immunoprecipitation (RIPA)lysis buffer and an SDS-PAGE gel configuration kit were purchased from Beyotime Institute of Biotechnology (Beijing, China). Luminata Classico Western HRP substrate and PVDF membrane were obtained from Millipore Corporation (Billerica, MA, USA). Rabbit anti-human CDK6 antibody was purchased from Beijing Bioss Biotechnology Ltd., (Beijing, China). Mouse anti-human β-actin was obtained from Santa Cruz Biotechnology Inc. (Santa Cruz, CA, USA).

The blank group consisted of untreated HeLa cells. The normal control (NC) group was transfected with the pcDNA6.2-GW plasmid. The miR-145 group was transfected with a pcDNA^TM^6.2-GW-miR-145 recombinant plasmid. The proliferation of HeLa cells was detected at 24, 48 and 72 h following transfection.

### Construction of miR-145 eukaryotic expression vector

Two types of single-stranded DNA were synthesized, one was: AATTCCACCTTGTCCTCACGGTCCAGTTTTCCCAGGAATCCCTTAGATGCTAAGATGGGGATTCCTGGAAATACTGTTCTTGAGGTCATGG, and the other was: AGCTTAACCATGACCTCAAGAACAGTATTTCCAGGAATCCCCATCTTAGCATCTAAGGGATTCCTGGGAAAACTGGACCGTGAGGACAAGG. The two single-stranded DNA sequences were annealed to synthesize double-stranded DNA, which was inserted into the pcDNA6.2-GW eukaryotic expression vector and amplified by *E. coli* DH5α.

### MTT assay

HeLa cells were cultured in Dulbecco’s modified Eagle’s medium (DMEM) supplemented with 10% fetal bovine serum. The cells were incubated in a thermostatic incubator at 37°C in an atmosphere with 5% CO_2_. The cells were seeded on a 96-well plate at a density of 3,000 cells/well and were transfected with liposomal transfection reagent (Roche). At 24, 48 and 72 h after transfection, cell proliferation was analyzed using MTT (Sigma Aldrich, St. Louis, MO, USA). Briefly, 20 μl MTT was added to the 96-well plate and incubated at 37°C for 4 h until purple precipitate was visible. Then, the culture supernatant was discarded and 150 μl dimethylsulfoxide was added. The 96-well plate was oscillated for 10 min until the purple precipitate was dissolved. The absorbance was measured at 492 nm on a microplate reader (Bio-Rad Laboratories, Hercules, CA, USA). Cell proliferation was calculated based on these absorbance values.

### Quantitative polymerase chain reaction (qPCR)

HeLa cells were cultured in DMEM supplemented with 10% fetal bovine serum. The cells were seeded on a 6-well plate at a density of 2–6×10^5^ cells/well and were transfected with liposomal transfection reagent. The RNA was extracted from these cells at 24 h after transfection using TRIzol. The PrimeScript^®^ RT kit was used for reverse transcription. The reverse primer was designed as: GTCGTATCCAGTGCGTGTCGTGGAGTCGGCAATTGCACTGGATACGACAGGGATT. The qPCR primers were as follows: forward, 5′-CAGTGCGTGTCGTGGAGT-3′; and reverse, 5′-AGGTCCAGTTTTCCCAGG-3′. U6 was selected as the internal standard. The CDK6 primers were as follows: forward, 5′-TGGAGACCTTCGAGCACC-3′; and reverse, 5′-CACTCCAGGCTCTGGAACTT-3′. β-actin was selected as the internal standard. SYBR Premix Ex Taq™ II was used for reaction. Briefly, the 20 μl qPCR system contained 1 μl reverse transcription product, 10 μl SYBR Premix Ex Taq^TM^ II, 1 μl forward primer (10 μM) and 1 μl reverse primer (10 μM). The reaction was performed on an FTC-3000 qPCR system (Funglyn Biotech Inc., Toronto, ON, Canada). The following PCR program was used: Pre-denaturation at 95°C for 1 min, followed by 40 cycles of 95°C for 10 sec and 60°C for 40 sec. The 2^−ΔΔCt^ method was used to quantify the expression of miR-145 and CDK6.

### Western blotting analysis

HeLa cells were cultured in DMEM supplemented with 10% fetal bovine serum. The cells were seeded on a 6-well plate at a density of 2–6×10^5^ cells/well and were transfected with liposomal transfection reagent. The proteins were extracted from these cells 24 h after transfection. According to the manufacturer’s instructions, the proteins were extracted with RIPA assay lysis buffer and examined by electrophoresis. Equal amounts of the proteins were separated on 10% SDS-PAGE and were electrophoretically transferred onto nitrocellulose membranes (Millipore Corporation), which were blocked in phosphate-buffered saline with Tween (PBST) containing 5% milk for 2 h. The film was incubated with primary antibody (anti-CDK6 1:100; anti-β-actin 1:3,000) at 4°C overnight. Following being washed with PBST and then incubated with the secondary antibody (HRP-conjugated goat anti-rabbit/mouse IgG; Pierce Biotechnology Inc., Rockford, IL, USA) at room temperature for another 1.5 h, the film was then washed with Tris-buffered saline and Tween-20, and the protein expression was visualized with chemiluminescence from the Luminata Classico Western HRP substrate.

### Statistical analyses

SPSS software, version 13.0 was used for data analysis (SPSS, Inc., Chicago, IL, USA). Comparisons of data between the groups were performed using a t-test. P<0.05 was considered to indicate a statistically significant difference.

## Results

### Successful construction of the miR-145 eukaryotic expression vector

**Figure 1:**
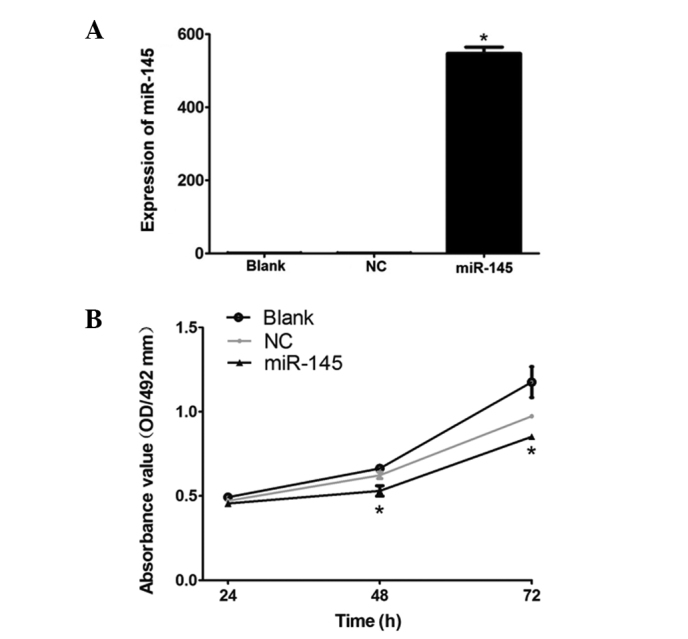
(A) mRNA expression level of miR-145 in each group. HeLa cells were divided into three groups, specifically, the untransfected blank group, empty vector group and miR-145 group, and were seeded into 6-well plates with a density of 2–6×10^5^ cells/well. After 24 h, quantitative polymerase chain reaction was employed to measure the expression levels of miR-145 in the three groups. ^*^P<0.01, statistically significant difference compared with the blank group. (B) Cell proliferation of each group. The three groups of HeLa cells were seeded into 96-well plates with a density of 3,000 cells/well. After 24, 48 and 72 h, the MTT assay was repeated three times to measure the cell proliferation of each group. ^*^P<0.05, statistically significant difference compared with the blank group (P<0.05). miR, microRNA; OD, optical density.

To identify whether miR-145 was expressed in the HeLa cervical cancer cells, qPCR was used. Data in Fig. 1A demonstrate that the expression level of miR-145 in the miR-145 group was significantly higher than that in the blank group (P=0.001), while no significant difference was observed in the expression level of miR-145 between the NC group and blank group (P=0.412). This demonstrates that the eukaryotic expression vector was successfully constructed and had a high transfection efficiency in the HeLa cells.

### miR-145 inhibits cervical cancer HeLa cell proliferation

To examine whether miR-145 inhibits HeLa cervical cancer cell proliferation 24, 48 and 72 h following transfection, an MTT assay was conducted. As demonstrated in Fig. 1B, the proliferation of miR-145-transfected HeLa cells was significantly lower than that of the blank group (P<0.05), whereas no marked difference was observed between the NC group and the blank group (P>0.05). This indicates that the high expression level of miR-145 inhibited the proliferation of HeLa cells.

### miR-145 inhibits the expression of CDK6

**Figure 2:**
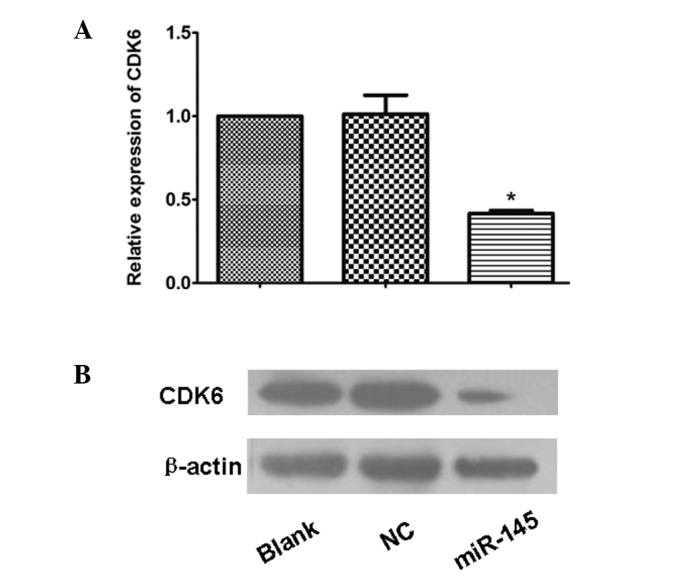
(A) The mRNA expression of CDK6 in each group of cells. HeLa cells were divided into three groups, specifically, the untransfected blank group, empty vector group and miR-145 group, and were seeded into 6-well plates with a density of 2–6×10^5^ cells/well. After 24 h, the total RNA was isolated from the three groups of cells and quantitative polymerase chain reaction was employed to measure the CDK6 mRNA expression level in each group. ^*^P<0.05, statistically significant difference compared with the blank group (n=3). (B) The protein expression of CDK6 in each group of cells. The three groups of HeLa cells were seeded into 6-well plates with a density of 2–6×10^5^ cells/well. After 24 h, the total protein was isolated from the three groups of cells and western blotting analysis was employed to detect the CDK6 protein expression in each group. CDK6, cyclin-dependent protein kinase 6; NC, normal control; miR, microRNA.

To investigate whether the overexpression of miR-145 in HeLa cells affects the expression of CDK6, qPCR and western blotting analysis were employed. Following transfection by the recombinant pcDNA6.2-GW-miR-145, the mRNA and protein levels of the downstream CDK6 in the HeLa cells were significantly lower than those in the NC and blank HeLa cells (P=0.001; Fig. 2). This demonstrates that miR-145 inhibited the mRNA and protein expression of CDK6 in the HeLa cervical cancer cells at the transcriptional and translational levels.

## Discussion

Abnormal miRNA expression is closely correlated with the occurrence and development of prostate (9), lung (10), colon (11) and numerous other human tumor types. Approximately half of the miRNA is located at tumor-associated genomic regions or fragile sites (12). The miRNA expression profiles of various tumor types are different (13). Currently, major miRNAs involved in tumor formation are divided into two categories. One category is oncogenic miRNA, which promotes tumorigenesis by inhibiting the expression of tumor suppressor genes, such as miR-106a (14) and miR-21 (15); the other category is tumor suppressor miRNA, which promotes tumor formation by activating oncogenes to inhibit cell differentiation and the cell cycle, such as miR-15b and miR-200b (16). At present, miRNA-overexpression vectors are commonly constructed to study the mechanisms of miRNAs and their downstream target genes in a variety of tumor types.

A number of studies have suggested that tumorigenesis may be caused by regulation disorders of cell cycle-related proteins, including cyclins, CDKs and CDK inhibitors. CDK6, including 7 exons, is one of the CDK family members located on human chromosome 7. It regulates the progress of the G1 phase by combining with cyclin D to promote the phosphorylation of the tumor suppressor gene, Rb, and by releasing the transcription factor, E2F, into the nucleus to affect the promoters of the associated genes (17), thereby promoting tumorigenesis.

miRNA expression profiles are different in various types of tumor. The same miRNA is able to regulate multiple target genes. For example, miR-21 targets both programmed cell death protein 4 (18) and myristoylated alanine-rich C kinase substrate (9). Similarly, the same gene may be regulated by a number of miRNAs. For instance, phosphatase and tensin homolog may be regulated by either miR-205 (10) or miR-21 (11). Therefore, it is very important to determine the corresponding downstream target genes for the late mechanism study of a particular tumor miRNA. The present study demonstrates that a good nucleotide complementary relationship exists between miR-145 and the CDK6 3′ untranslated region, by revealing that the expression of CDK6 is significantly inhibited by miR-145 transfection. This suggests that miR-145 may directly target the expression of CDK6 to inhibit the proliferation of cervical cancer cells.

In summary, the expression of pcDNA6.2-GW-miR-145 downregulated the expression of CDK6 and inhibited the proliferation of HeLa cervical cancer cells, providing a basis for the further study of the effects of miR-145 on the biological behaviors of cervical cancer cells and the associated mechanisms.
